# The association of plant or animal food origin and processing level with BMI and waist circumference: a prospective cohort study of the UK Biobank

**DOI:** 10.1016/j.eclinm.2026.104050

**Published:** 2026-07-02

**Authors:** Patrícia de Fragas Hinnig, Ana Giulia Forjaz Grassi, Kiara Chang, Eszter P. Vamos, Renata Bertazzi Levy, Fernanda Rauber

**Affiliations:** a Post-Graduation Program in Nutrition, Health Sciences Centre, Federal University of Santa Catarina, Florianopolis 88040-900, Brazil; b Department of Nutrition, School of Health, Sciences, University of Brasília (UnB), Brasília, DF, Brazil; c Department of Preventive Medicine, School of Medicine, University of São Paulo, São Paulo 01246-903, Brazil; d Public Health Policy Evaluation Unit, School of Public Health, Imperial College London, London W12 0BZ, UK; e Centre for Epidemiological Studies in Health and Nutrition, University of São Paulo, São Paulo 01246-904, Brazil; f Institute of Biomedical Research of Salamanca (IBSAL), University of Salamanca, Salamanca 37007, Spain

**Keywords:** BMI, Abdominal obesity, Plant food, Animal food, Ultra-processed foods

## Abstract

**Background:**

Body Mass Index (BMI) and waist circumference (WC) gain are key risk factors for cardiometabolic disease and mortality. Although plant-based diets are often promoted for health and sustainability, the rising inclusion of ultra-processed foods (UPF) within these diets raises concerns. This study assessed the associations of food origin (plant vs. animal) and processing level (UPF vs. non-UPF) with BMI and WC increase in a prospective cohort.

**Methods:**

A total of 17,374 participants aged 40–69 years from the UK Biobank cohort study were included. Dietary data were collected between 2009 and 2012, and follow-up assessments were conducted between 2012 and 2019. Dietary intake was assessed using a minimum of two and a maximum of five repeated 24-h dietary recalls. Foods were classified by plant or animal origin and processing level using the Nova system. Outcomes were defined separately for BMI and WC as increases of ≥5% and ≥10% from baseline to follow-up. Cox proportional hazards models were applied.

**Findings:**

During a mean follow-up of 5.3 years, 2477 cases of ≥5% BMI increase and 4567 of ≥5% WC increase were observed. A 10% higher energy intake from plant-sourced non-UPF was associated with 11% lower hazard of ≥5% BMI increase (HR 0.89, 95% CI 0.85–0.92) and 10% lower hazard of ≥5% WC increase (HR 0.90, 95% CI 0.88–0.93). A 10% higher intake of plant-sourced UPF was linked to increased risk of both BMI and WC increase (HR 1.10 and 1.09, respectively). Substitution analyses indicated that a higher contribution of plant-sourced non-UPF, at the expense of UPF (plant or animal), was consistently associated with a lower risk of study outcomes.

**Interpretation:**

Our findings highlight the importance of considering food origin alongside processing, showing that plant-sourced non-UPF may confer protective effects, whereas plant-sourced UPF contribute to adverse weight outcomes. Future research is needed to clarify the behavioural and biological mechanisms behind these findings.

**Funding:**

This research received funding from World Cancer Research Fund (WCRF UK), as part of the World Cancer Research Fund International grant programme.

**Research in context**

**Evidence before this study**

To establish the existing evidence, we searched PubMed, Embase, Web of Science, and Scopus from database inception to August 31, 2024, for prospective studies on the associations between plant-based diets, ultra-processed foods (UPF), and changes in body mass index (BMI) or waist circumference. Our search terms included combinations of “plant-based diet,” “ultra-processed foods,” “Nova,” “body mass index,” “waist circumference,” and “weight gain.” We focused on observational studies in adults that reported longitudinal changes in adiposity, excluding cross-sectional analyses.

The existing literature consistently links higher UPF intake to weight gain and abdominal obesity, while associating plant based diets with more favourable weight trajectories. However, most prior studies have examined plant-based diets and UPF in isolation. Very few have jointly considered both food’s origin (plant vs. animal) and degree of processing. Notably, we identified a gap: no studies were found that directly assessed the combined and substitutive effects of plant-sourced UPF and plant-sourced non-UPF on long-term changes in BMI and waist circumference.

**Added value of this study**

To our knowledge, this is the first large prospective study to simultaneously account for food origin and processing level in relation to long-term changes in BMI and waist circumference. Over a mean follow-up exceeding five years, we found that a higher intake of plant-sourced non-UPF is associated with a lower risk of increases in both BMI and waist circumference. Conversely, a higher intake of plant-sourced UPF was associated with an elevated risk. A key finding from our substitution analyses is that replacing any UPF—whether plant- or animal-sourced—with plant-sourced non-UPF consistently reduces the risk of adverse weight outcomes. These results underscore a critical nuance: not all plant-sourced foods confer equal metabolic benefits, and the level of processing plays a critical role.

**Implications of all the available evidence**

Taken together with existing literature, our results suggest that public health recommendations should move beyond broad promotion of plant-based diets and explicitly emphasise minimally processed plant foods while discouraging UPF, including those marketed as plant-based. These findings support dietary guidelines that integrate both food origin and processing and may inform strategies aimed at preventing weight gain and abdominal obesity. Future research is needed to clarify the behavioural and biological mechanisms behind these findings.

## Introduction

Weight gain and increases in waist circumference (WC), as indicators of adiposity, are associated with a higher risk of coronary heart disease and mortality.^1^^,^^2^ Evidence from different populations indicates that both excess weight and abdominal adiposity have increased over recent decades, with important implications for public health.^3^^,^^4^ These trends are likely driven by broader changes in the food environment and food systems, particularly the growing availability, accessibility, and marketing of ultra-processed foods (UPFs).^5^

In this context, plant-based diets, which involve reducing or eliminating the consumption of eggs, dairy products, poultry, fish, and meat, have attracted growing attention due to their potential benefits for health, weight management, animal welfare, and environmental sustainability.^6^ Although such dietary patterns have existed for centuries, their popularity has surged in recent years. In the United Kingdom (UK), for example, the proportion of individuals consuming plant-based alternative foods increased from 6.7% in 2008–2011 to 13.1% in 2017–2019.^7^

Evidence suggests that plant-based diets are associated with a lower risk of cardiovascular disease, hypertension, type 2 diabetes, and certain cancers,^8^^,^^9^ and intervention studies indicate that transitioning to a plant-based diet may contribute to weight reduction.^10^ However, emerging evidence highlights an important nuance: modern plant-based dietary patterns, including vegetarian and vegan diets, often incorporate a higher proportion of UPFs.^11^^,^^12^ In the UK, vegetarians have been shown to consume more UPFs than regular meat eaters, whereas moderate meat/fish consumers tend to have lower UPF intake.^11^ Higher UPF consumption has been consistently associated with higher body mass index (BMI), greater WC, increased obesity risk, and a range of other adverse health outcomes.^13^, ^14^, ^15^, ^16^, ^17^ Despite the growing popularity of plant-based diets, the health implications of consuming plant-sourced UPFs, particularly regarding weight and WC, remain poorly understood.

Evidence from a cohort of British adults suggests that food processing and food origin may differentially influence cardiovascular risk. For every 10-percentage-point increase in the consumption of plant-sourced non-UPF, a 7% lower risk of cardiovascular disease (CVD) and a 13% lower risk of CVD mortality were observed, whereas higher consumption of plant-sourced UPF was associated with a 5% higher risk of CVD and a 12% increase in CVD mortality.^15^ These findings underscore the need to investigate the combined effects of food origin (plant- vs. animal-based) and processing level on weight and WC gain, which, to date, have not been examined in any study. Accordingly, this study aims to evaluate the associations between the dietary contribution of different food groups, considering both origin and processing level, and subsequent changes in body weight and WC using data from the UK Biobank Prospective Cohort. We hypothesised that plant-sourced UPFs were associated with increased weight and WC gain.

## Methods

### Study design and participants

The UK Biobank is a prospective cohort study of over 500.000 adults aged 40−69 years recruited between 2006 and 2010 across England, Scotland, and Wales. Baseline assessment was conducted at recruitment (2006–2010). Dietary data were collected between 2009 and 2012. Follow-up assessments were conducted in 2012–2013 and 2014–2019, during which outcome measures were obtained.

At baseline and follow-up assessments, participants completed a self-administered touch-screen questionnaire on socio-demographic, lifestyle and health-related data, trained staff collected physical and anthropometric measurements using standardized procedures. Detailed descriptions of study measures are available in the UK Biobank online protocol (http://www.ukbiobank.ac.uk).

### Ethics

The UK Biobank received ethical approval from the North West Multi-Centre Research Ethics Committee (21/NW/0157), and all participants provided written informed consent.

### Procedures

Dietary intake was evaluated using a validated, self-administered, web-based 24-h dietary recall questionnaire, administered on up to five occasions between 2009 and 2012. For these analyses, food consumption was classified according to the proportion of total energy derived from plant- vs. animal-based foods. These categories were further stratified based on the contribution of UPF vs. non-UPF to total energy intake.

Plant-origin foods were defined as those primarily or exclusively sourced from plants, including fruits, vegetables, grains, and breads. Animal-derived foods encompassed all meats (e.g., poultry, fish, and red meats), dairy products, and eggs. The classification of food processing was based on the Nova system, which categorizes foods according to the degree and purpose of industrial processing.^18^ This system consists of four main groups: (1) unprocessed or minimally processed foods (e.g., fresh, dried, or frozen fruits and vegetables; grains; pasta; plain milk; plain yogurt; fresh or frozen meats); (2) processed culinary ingredients (e.g., table sugar, oils, butter, and salt); (3) processed foods (e.g., canned vegetables, cheese, simple breads, fruit in syrup, canned fish); and (4) UPFs (e.g., sugar-sweetened beverages, packaged snacks, confectionery, packaged breads, reconstituted meat products, and ready-to-eat frozen or shelf-stable meals). In this study, we estimated the proportion of total dietary energy from UPF relative to non-UPFs, with non-UPF encompassing the first three Nova groups. Supplementary Table S1 details examples of food items considered in each food group, considering both food origin and processing.

Food classification followed previously described approaches for both food origin and processing level using UK Biobank data.^13^^,^^15^ When foods were not available in a disaggregated form, classification was based on the most commonly consumed formulation in the UK population, informed by national dietary data. The approach for categorizing plant-sourced and animal-derived foods,^15^ as well as the assignment of food items to Nova categories based on 24-h dietary recall data,^13^ has been previously detailed in the literature.

The dietary contributions of plant-sourced non-UPF, plant-sourced UPF, all plant-sourced foods, and total UPF consumption were analysed both as quartiles and as continuous variables (per 10% increment in energy contribution).

### Outcomes assessment

Outcomes were assessed using BMI and WC. Height, weight, and WC were measured by trained fieldworkers using standardized procedures, with height obtained using a portable stadiometer and weight using calibrated weighing scales.^19^ Measurements were collected at baseline and repeated during follow-up assessments. BMI was computed as weight (kg) divided by height (m) squared (kg/m^2^). WC was measured to the nearest 0.1 cm at the midpoint between the iliac crest and the lower rib. To evaluate changes over time, we derived four binary outcomes indicating whether participants had a ≥5% or ≥10% increase in BMI and WC from baseline to follow-up.^13^^,^^20^, ^21^, ^22^

### Covariates

Baseline study covariates included: age, sex (male, female), ethnicity (white, non-white), baseline BMI or WC (continuous, depending on the outcome), physical activity (low, moderate, high, missing), smoking status (never previous, current), Index of Multiple Deprivation (IMD; quintile), and region (London, South East, South West, East Midlands, West Midlands, Yorkshire & the Humber, North East, North West, Wales, Scotland). IMD is a composite measure of deprivation for each small area of the UK based on participants’ postcode, and we derived IMD quintiles based on deprivation scores.

Participants with missing covariates data were excluded, except for physical activity and IMD variables. Since 2236 (12.9%) and 362 (2.1%) participants had missing data on physical activity and IMD variables, respectively, we included a missing class into the models for these variables to preserve sample size.

The selection of potential confounders was guided by an extensive literature review and conceptual reasoning, focusing on variables that have been consistently associated with both the exposure and the outcome.

### Statistical analyses

**Fig. 1:**
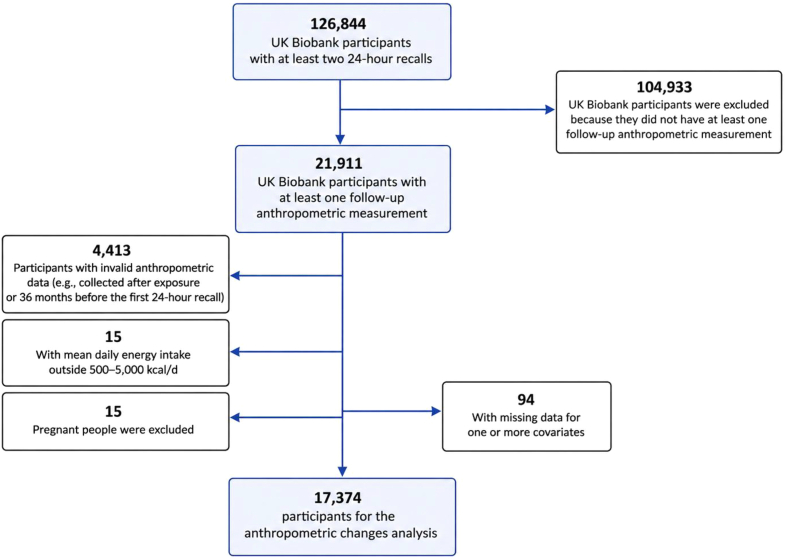
Study profile.

For this study, we analysed a subsample of 21,911 UK Biobank participants who had completed at least two 24-h dietary recalls and had at least one follow-up anthropometric measurement. We excluded participants who did not meet the inclusion criteria regarding the temporal alignment between exposure assessment and anthropometric measurements at baseline and follow-up (n = 4413). For example, participants were excluded when the exposure assessment occurred at a time point excessively distant from the baseline anthropometric measurement. We also excluded participants with a total daily energy intake outside of the predefined limits (<500 kcal and >5000 kcal)^23^ (n = 15), women who were pregnant at baseline or became pregnant during the follow-up period (n = 15), and participants with missing data for one or more covariates (n = 94). Data from 17,374 participants were included in the analyses (Fig. 1).

We characterized the study population at baseline and across quartiles of dietary energy contribution from plant-sourced non-UPF. Differences between quartiles were assessed using analysis of variance (ANOVA) for continuous variables and χ^2^ tests for categorical variables, as appropriate.

We evaluated differences in survival distributions among subgroups using log-rank tests. To estimate hazard ratios and their corresponding 95% confidence intervals for each outcome across quartiles of food contribution, using the lowest quartile as the reference (or as a continuous variable, as previously described). All participants had a baseline measure of the outcome. We applied Cox proportional hazards regression models, considering age as the underlying time scale. Age at recruitment was used as the entry time, and participants were followed up until the age at the occurrence of the outcome or their last assessment, whichever came first. Participants who did not develop the outcome were censored at their last follow-up assessment. The models were adjusted for sex, ethnicity, baseline BMI or WC (depending on the outcome), physical activity, smoking status, IMD, and geographic region. The proportional hazards assumption was assessed by examining Schoenfeld residuals in relation to survival time. This analysis indicated a violation of the proportionality assumption for sex in both outcomes and for baseline WC in relation to WC increase, which led us to stratify these variables in the models. The outcomes of having a ≥5% and ≥10% increase in BMI and WC were analysed in separate models, and participants who reached a ≥5% increase were not censored in the ≥10% model. Linear trends were examined across quartiles. To assess the assumption of linearity between food group intake and the risk of BMI or WC increase, we employed restricted cubic spline functions.

Given that the four dietary variables analysed (plant-sourced non-UPF, plant-sourced UPF, animal-sourced non-UPF, and animal-sourced UPF) represent compositional data based on percentage intake, we conducted a relative substitution analysis. Specifically, to examine the impact of replacing 10% of each of the three presumably less healthy food groups (animal-sourced food groups and plant-sourced UPF) with 10% of plant-sourced non-UPFs on the risk of BMI or WC increase, we used Cox proportional hazards regression models. In these models, three food groups were included simultaneously, while the fourth served as the reference category. The resulting risk estimates reflected the association of every 10% increment in one of the three food groups relative to an equivalent proportion of plant-sourced non-UPFs. These estimates reflect relative differences in dietary composition and arise from the specific way the model is specified, rather than representing a single or directly observable substitution effect. This analysis was performed using the same covariate adjustments as in the main models.

The following sensitivity analyses were also performed: (i) additionally adjusting for free sugars (% of total energy), saturated fat (% of total energy), sodium density (mg/1000 kcal), and fibre density (g/1000 kcal), to account for overall diet quality and evaluate whether associations are independent of nutrient composition; (ii) considering food groups as a proportion of daily grams intake (% of total grams) and additionally adjusting for total daily energy intake (kcal/day), to assess robustness to alternative exposure definitions and to capture UPFs with low or no energy contribution (e.g., artificially sweetened beverages); (iii) excluding participants who self-reported a low-calorie diet at baseline (2.8%), to reduce potential reverse causation and residual confounding; (iv) excluding alcoholic beverages from the UPF group and additionally adjusting for alcohol intake (g/day), to ensure that associations are not driven by alcohol consumption, given its distinct behavioural and metabolic profile.

As a post-hoc analysis added during peer review, we additionally evaluated the following sensitivity analysis: v) excluding participants with severe obesity (BMI ≥ 40 kg/m^2^) in baseline to minimize the potential for residual confounding; vi) excluding participants with diabetes in baseline to minimize the potential for residual confounding. Also, we assessed potential effect modification by baseline BMI and WC by testing interaction terms with the main exposures and formally tested for interaction between sex and the main exposures.

All statistical analyses were conducted using Stata version 14.0 and a p-value of <0.05 was considered statistically significant.

### Role of the funding source

The funding agencies listed in the funding section had no involvement in study design, data collection, data analyses, data interpretation, or the writing of the report.

## Results

Among the 17,374 participants (52.3% female), the mean age at baseline was 56.1 ± 7.4 years. Table 1 presents the main baseline characteristics of participants, categorized by quartiles of plant-sourced non-UPF contribution to the diet. Compared to those in the lowest quartile, participants in the highest quartile were, on average, older, more likely to be female, non-white, and ex- or current smokers. They also had a lower mean BMI, higher physical activity levels, and were more likely to live in less deprived areas. Regarding nutrient intake, participants in the highest quartile tended to consume less total energy, free sugars, saturated fats, and sodium, while having a higher fibre intake compared to those in the lowest quartile (Table 1).

**Table 1:** Characteristics of the study population according to quartiles of the dietary contribution of plant-sourced non-ultra-processed foods, UK Biobank cohort (n = 17,374).

	All participants	Quartile of the dietary contribution of plant-sourced non-UPF (% of total energy)				p value
		1 (16.2%)	2 (25.9%)	3 (33.6%)	4 (46.0%)	
	mean (SD) or % (n)					
Baseline age, years (mean, SD)	56.1 (7.4)	55.3 (7.7)	56 (7.4)	56.4 (7.3)	56.6 (7.1)	<0.001
Female sex (%, n)	52.3 (9088)	50.8 (2208)	54.1 (2351)	52 (2259)	52.3 (2270)	0.021
Male sex (%, n)	47.7 (8286)	49.2 (2136)	45.9 (1992)	48 (2085)	47.7 (2073)	
Ethnicity White (%, n)	97.9 (17,006)	98.5 (4278)	98.1 (4262)	97.9 (4251)	97.1 (4215)	<0.001
Baseline BMI, kg/m^2^ (mean, SD)	26.6 (4.3)	27.2 (4.7)	26.6 (4.3)	26.3 (4.1)	26.1 (3.9)	<0.001
Baseline WC, cm (mean, SD)	88.2 (12.9)	90 (13.4)	88.1 (12.8)	87.6 (12.6)	87.2 (12.4)	<0.001
Physical activity (%, n)						
Low	16.3 (2829)	19.8 (862)	17 (738)	15.3 (664)	13 (565)	<0.001
Moderate	37 (6429)	35.3 (1534)	36.7 (1593)	38.5 (1671)	37.6 (1631)	
High	33.8 (5880)	29.8 (1293)	32.9 (1430)	33.8 (1470)	38.8 (1687)	
Missing	12.9 (2236)	15.1 (655)	13.4 (582)	12.4 (539)	10.6 (460)	
Smoking status (%, n)						
Never smoked	61.3 (10,655)	66.9 (2906)	61.5 (2672)	60.8 (2641)	56.1 (2436)	<0.001
Ex-smoker	33.4 (5802)	28 (1216)	33.3 (1445)	34.2 (1486)	38.1 (1655)	
Current smoker	5.3 (917)	5.1 (222)	5.2 (226)	5 (217)	5.8 (252)	
Index of Multiple Deprivation (%, n)						
1st quintile (least deprived)	19.1 (3310)	15.9 (692)	20.8 (902)	19.8 (859)	19.7 (857)	<0.001
2nd quintile	19.5 (3379)	17.8 (772)	19.7 (854)	20.2 (876)	20.2 (877)	
3rd quintile	20.7 (3599)	21.2 (920)	20.8 (904)	20.7 (899)	20.2 (876)	
4th quintile	19.8 (3433)	20.7 (900)	19.1 (829)	19.1 (828)	20.2 (876)	
5th quintile (most deprived)	18.9 (3291)	22.3 (969)	17.5 (758)	18.2 (791)	17.8 (773)	
Missing	2.1 (362)	2.1 (91)	2.2 (96)	2.1 (91)	1.9 (84)	
Geographical region (%, n)						
London	4.7 (822)	2.7 (115)	4.1 (179)	4.7 (205)	7.4 (323)	<0.001
South East	1.8 (318)	1.6 (68)	1.9 (83)	1.8 (78)	2.1 (89)	
South West	0.3 (54)	0.3 (11)	0.4 (18)	0.3 (14)	0.3 (11)	
East Midlands	8.6 (1487)	8.5 (371)	8.3 (360)	9.3 (404)	8.1 (352)	
West Midlands	10.1 (1750)	10.3 (448)	10.2 (441)	9.8 (427)	10 (434)	
Yorkshire & the Humber	34.1 (5929)	33.1 (1438)	34 (1477)	35.3 (1533)	34.1 (1481)	
North East	11 (1914)	12.5 (541)	10.9 (474)	11.1 (480)	9.7 (419)	
North West	28.5 (4959)	30.6 (1330)	29.3 (1272)	26.9 (1169)	27.4 (1188)	
Wales	0.3 (47)	0.2 (7)	0.4 (18)	0.2 (10)	0.3 (12)	
Scotland	0.5 (94)	0.4 (15)	0.5 (21)	0.6 (24)	0.8 (34)	
Nutrients (mean, SD)						
Total energy (kcal)	2060 (533)	2197 (596)	2104 (518)	2030 (503)	1911 (464)	<0.001
Free sugars (% of energy)	13.6 (6.4)	15.7 (7.3)	14.3 (6.2)	12.9 (5.7)	11.6 (5.4)	<0.001
Saturated fats (% of energy)	10.8 (3)	12.5 (2.9)	11.3 (2.7)	10.5 (2.6)	9.1 (2.6)	<0.001
Sodium (mg/1000 kcal)	936 (210)	1020 (213)	954 (192)	913 (193)	855 (205)	<0.001
Fibre (g/1000 kcal)	12.6 (4.3)	11.5 (4.1)	12.2 (4.0)	12.7 (4.0)	13.9 (4.8)	<0.001

BMI = Body Mass Index; WC = waist circumference; UPF = ultra-processed foods. Comparisons between groups were performed using analysis of variance or χ2 tests, as appropriate.

The average contribution of plant-souced foods to total daily energy intake was 69.9%, with 30.1% coming from non-UPF and 39.8% from UPF. As for the remaining diet, 21.2% was derived from animal-sourced non-UPF, while 8.8% came from animal-sourced UPF (Table 2).

**Table 2:** Average dietary contribution (% of total energy intake) of foods grouped according to both plant or animal origin and food processing categories.

Plant-sourced foods			Animal-sourced foods		
	%	SD		%	SD
**Non-ultra-processed**	**30**.**1**	**11**.**4**	**Non-ultra-processed**	**21**.**2**	**8**.**4**
Fruit	8.8	5.5	Red meat^b^	4.6	4.7
Beer and Wine	5.9	6.9	Milk	4.4	3.6
Cereals	3.6	4.4	Fish	3.1	4.7
Vegetables	2.5	1.8	Cheese	3.0	3.1
Pasta	2.1	3.6	Poultry	2.4	3.1
Roots and tubers	1.7	2.0	Animal fats	2.1	3.3
Processed bread	1.6	3.2	Eggs	1.6	2.5
Nuts and seeds	1.2	2.4			
Table sugar	0.8	2.1			
Vegetables/fruit preserved	0.7	0.9			
Legumes	0.6	1.4			
Others^a^	0.5	1.2			
**Ultra-processed**	**39**.**9**	**12**.**9**	**Ultra-processed**	**8.8**	**8.0**
Industrialised packaged breads	10.3	5.8	Milk-based drinks	4.3	6.8
Pastries, buns, and cakes	7.1	6.8	Sausage and other reconstituted red meat products^b^	1.5	3.0
Biscuits	3.8	4.5			
Margarine and other spreads	3.3	2.9	Nuggets and other reconstituted meat products	1.3	2.9
Industrial chips (French fries)	2.8	3.7			
Confectionery	2.6	3.5	Milk based desserts	1.0	1.6
Breakfast cereals	2.8	3.2	Mayonnaise and spreadable cheese	0.7	1.5
Soft drinks, fruit drinks, and fruit juices	2.0	3.1			
Packaged salty snacks	1.6	2.5			
Industrial pizza	1.3	4.7			
Packaged pre-prepared meals	0.9	1.5			
Alcoholic drink	0.7	2.2			
Sauces, dressing and gravies	0.3	0.5			
Meat alternatives	0.2	1.0			
**Total**	**69**.**9**	**10**.**2**	**Total**	**30**.**1**	**10**.**2**

UK Biobank cohort (n = 17,374).

a Coffee and tea, fungi, homemade soup, plant oil.

b Considered as red meat in the analyses using non-red meat vs. red meat, according to degree of food processing.

### UPF, plant-sourced foods and BMI and WC increase

The associations between the dietary contribution of food groups, considering both plant or animal origin and food processing categories (% of total energy), and the risks of having a ≥5% and ≥10% increase in BMI and WC are shown in Table 3. No violations of linearity were detected (Supplementary Fig. S1 and Table S2).

**Table 3:** Association between the consumption of foods, with a focus on food processing, and BMI or WC increase in the UK Biobank cohort (n = 17,374).

	Consumption (% of total energy)													
	Quartile^a^													Continuous (10% increase in the consumption)
	1		2			3			4					
	Hazard ratio (HR) (95% CI)										p for trend			Hazard ratio (HR) (95% CI)
**For having a ≥ 5% BMI increase**														
n for cases/non-cases = 2477/14,897														
Plant-sourced non-UPF	**1**	**0.85**	0.76	0.95	**0.76**	0.68	0.84	**0.69**	0.62	0.78	<0.001	**0.89**	0.85	0.92
Plant-sourced UPF	**1**	**1.06**	0.94	1.19	**1.16**	1.03	1.30	**1.34**	1.20	1.50	<0.001	**1.10**	1.06	1.13
Total plant-sourced	**1**	**1.02**	0.91	1.14	**0.99**	0.88	1.10	**1.02**	0.91	1.14	0.916	**1.00**	0.96	1.04
Total UPF	**1**	**1.02**	0.91	1.15	**1.19**	1.06	1.33	**1.37**	1.23	1.54	<0.001	**1.10**	1.07	1.13
**For having a ≥ 10% BMI increase**														
n for cases/non-cases = 737/16,637														
Plant-sourced non-UPF	**1**	**0.83**	0.68	1.00	**0.66**	0.54	0.81	**0.63**	0.51	0.77	<0.001	**0.85**	0.80	0.91
Plant-sourced UPF	**1**	**0.97**	0.78	1.20	**1.17**	0.95	1.44	**1.44**	1.17	1.77	<0.001	**1.13**	1.07	1.19
Total plant-sourced	**1**	**0.91**	0.74	1.12	**0.90**	0.73	1.10	**1.06**	0.87	1.29	0.635	**1.00**	0.93	1.08
Total UPF	**1**	**1.00**	0.80	1.25	**1.32**	1.07	1.63	**1.53**	1.24	1.88	<0.001	**1.13**	1.07	1.20
**For having a ≥ 5% WC increase**														
n for cases/non-cases = 4567/12,807														
Plant-sourced non-UPF	**1**	**0.89**	0.82	0.97	**0.81**	0.74	0.88	**0.74**	0.68	0.81	<0.001	**0.90**	0.88	0.93
Plant-sourced UPF	**1**	**1.09**	1.00	1.19	**1.14**	1.04	1.24	**1.37**	1.25	1.49	<0.001	**1.09**	1.06	1.11
Total plant-sourced	**1**	**1.02**	0.94	1.11	**1.05**	0.96	1.14	**1.02**	0.94	1.11	0.498	**1.00**	0.98	1.03
Total UPF	**1**	**1.07**	0.98	1.17	**1.18**	1.08	1.29	**1.36**	1.25	1.49	<0.001	**1.09**	1.06	1.11
**For having a ≥ 10% WC increase**														
n for cases/non-cases = 1908/15,466														
Plant-sourced non-UPF	**1**	**0.88**	0.78	1.00	**0.72**	0.64	0.82	**0.62**	0.54	0.72	<0.001	**0.85**	0.82	0.89
Plant-sourced UPF	**1**	**1.11**	0.97	1.27	**1.19**	1.03	1.36	**1.54**	1.34	1.75	<0.001	**1.13**	1.09	1.17
Total plant-sourced	**1**	**1.01**	0.88	1.15	**0.98**	0.86	1.12	**1.02**	0.90	1.16	0.863	**1.00**	0.95	1.04
Total UPF	**1**	**1.03**	0.90	1.18	**1.28**	1.12	1.47	**1.48**	1.30	1.70	<0.001	**1.13**	1.09	1.17

BMI = Body Mass Index; WC = waist circumference; UPF = ultra-processed foods. Values in bold represent hazard ratios. Mean follow-up times were 5.3 for BMI increase (92,129,583 person-years) and for WC increase (92,135,083 person-years).

a Cut-offs for quarters of food consumption ranged from 16.3% (1st quartile) to 45.2% (4th quartile) for plant-sourced foods non-UPF; from 23.5% to 56.5% for plant-sourced UPF; from 56.6% to 82.0% for total plant-sourced foods; and 31.2%–65.9% for total UPF, respectively. Cox proportional hazards models with age as the underlying timescale. Adjusted by sex, ethnic (white, non-white), baseline BMI or WC (continuous, depending on the outcome), physical activity (low, moderate, high, missing), smoking status (never, previous, current), index of multiple deprivation (quintile), and region (London, South East, South West, East Midlands, West Midlands, Yorkshire & the Humber, North East, North West, Wales, Scotland).

A total of 2477 cases of having a ≥5% increase in BMI and 737 cases of having a ≥10% increase in BMI occurred during 92,129,583 person-years of follow-up (mean, 5.3 years). After adjustment for potential confounders, a 10% increase in the contribution of plant-sourced non-UPF in the diet was associated with an 11% lower hazard of having a ≥5% increase in BMI (HR 0.89; 95% CI 0.85–0.92) and a 15% lower hazard of a ≥10% increase in BMI (HR 0.85; 95% CI 0.80–0.91). In contrast, plant-sourced UPF contribution was associated with an increased risk of both outcomes (adjusted HR 1.10; 95% CI 1.06–1.13 for a ≥5% increase; and adjusted HR 1.13; 95% CI 1.07–1.19 for a ≥10% increase). A higher dietary contribution of UPF overall was associated with an increased risk of all outcomes related to BMI increase (HR for a 10% increase in the contribution: 1.10; 95% CI 1.07–1.13 for a ≥5% increase; HR 1.13; 95% CI 1.07–1.20 for a ≥10% increase), while there was no evidence of an association between the total plant-sourced food contribution and any outcomes (Table 3).

A total of 4567 cases of ≥5% increase and 1908 cases of ≥10% increase in WC occurred during 92,135,083 person-years of follow-up (mean, 5.3 years). After adjustment for potential confounders, a 10% increase in the dietary contribution of plant-sourced non-UPF was associated with a 10% lower hazard of having a ≥5% increase in WC (HR: 0.90; 95% CI 0.88–0.93) and a 15% lower hazard of having a ≥10% increase in WC (adjusted HR 0.85; 95% CI 0.82–0.89). Meanwhile, plant-sourced UPF contribution was associated with a higher risk (HR 1.09; 95% CI 1.06–1.11 for a 5% increase; and HR 1.13; 95% CI 1.09–1.17 for a 10% increase) (Table 3).

A higher dietary contribution of UPF overall was associated with an increased risk of all outcomes related to WC increase (HR for a 10% increase in the contribution: 1.09; 95% CI 1.06–1.11 for a 5% increase; HR 1.13; 95% CI 1.09–1.17 for a 10% increase), while there was no evidence of an association with the total plant-sourced food contribution (Table 3).

The analysis based on quartiles of dietary contribution revealed trends that were consistent with the results from the analysis using continuous variables (Table 3).

**Fig. 2:**
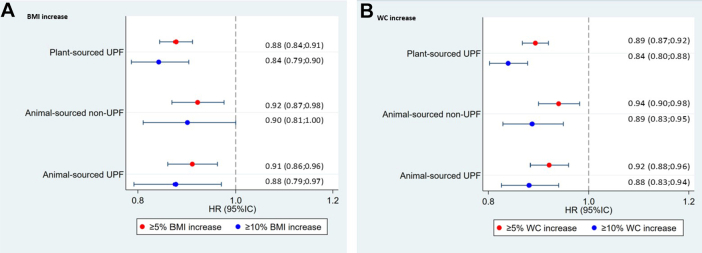
Effect of replacing 10% of each of the three food groups (plant-sourced UPF, animal-sourced non-UPF, and animal-sourced UPF) with plant-sourced non-UPF on BMI (Fig. 2A) and WC (Fig. 2B) increases. Fully adjusted hazard ratios (HRs) and 95% confidence intervals (CIs) were estimated using Cox proportional hazards regression models. All results were derived from continuous linear models. BMI = body mass index; WC = waist circumference; UPF = ultra-processed foods.

In our substitution analysis (Fig. 2), a 10-percentage-point higher contribution of plant-sourced non-UPFs, at the expense of plant-sourced UPF, animal-sourced non-UPF, or animal-sourced UPF, was associated with a lower risk of ≥5% and ≥10% increases in BMI and WC.

### Additional analyses and further adjustments

The analysis based on the dietary contribution of total grams was generally consistent with the main findings (Supplementary Table S3), with the association for total plant-sourced foods shifting towards an inverse and statistically significant relationship with both ≥5% and ≥10% increases in BMI and WC.

Sensitivity analyses including additional adjustments for free sugars saturated fat, sodium density, and fibre showed similar results. However, the association between a 10% increase in total UPF consumption and the risk of a ≥10% increase in BMI lost statistical significance (Supplementary Table S4).

Additional analyses excluding participants on low-calorie diets, and alcoholic beverages yielded results consistent with the main analysis (Supplementary Tables S5 and S6). Additionally, the results remained similar to those of the main analysis in sensitivity analyses excluding participants with severe obesity at baseline (Supplementary Table S7) and those with diabetes at baseline (Supplementary Table S8).

We assessed potential effect modification by baseline BMI and WC by testing interaction terms with the main exposures, and found no evidence of interaction (data not shown). We also formally tested for interaction between sex and the main exposures, and no evidence of effect modification was observed (Supplementary Table S9).

## Discussion

In this large population-based prospective cohort, we found that a higher dietary contribution of plant-sourced non-UPF was associated with a lower risk of both BMI increase and WC increase, whereas plant-sourced UPF intake was associated with increased risk of these outcomes over a mean follow-up of 5.3 years. Importantly, higher consumption of plant-sourced non-UPF at the expense of plant-sourced UPF was associated with an approximately 12–16% lower hazard of moderate and large BMI increase, and 10–15% lower hazard of moderate and large WC increase, respectively. Given that a substantial proportion of participants experienced clinically meaningful increases in BMI and WC during follow-up, these reductions in risk highlight the public health relevance of emphasizing minimally processed plant foods. Overall, our findings highlight that the protective associations of plant-sourced foods are contingent on their level of processing.

Observational studies have consistently reported longitudinal associations between UPF consumption and weight gain or WC increase.^13^^,^^15^^,^^22^^,^^24^ While most of the available evidence does not distinguish UPF by plant or non-plant origin, these concerns gain additional relevance with the rapidly growing market of plant-sourced UPF.^25^ Supporting this, a UK Biobank analysis showed that the contribution of UPF was higher among vegetarian diets compared with diets containing modest amounts of meat or fish,^11^ suggesting that shifts toward plant-based eating patterns may inadvertently increase UPF exposure.

Our results complement and extend previous evidence on the role of food processing in shaping the health impacts of plant-sourced foods. Recent cohort study, including analyses from the UK Biobank^15^ consistently found that higher consumption of plant-sourced non-UPF was associated with lower risk of CVD incidence and mortality, whereas greater intake of plant-sourced UPF was linked to higher risk of these outcomes. By focusing on weight gain and abdominal adiposity, our study provides novel evidence that the level of processing also modifies the associations between plant-sourced food intake and intermediate outcomes closely linked to cardiometabolic health outcomes.

The differential associations observed for plant-sourced non-UPF and UPF with weight and WC gain are likely multifactorial. Although both originate from plant sources, they differ markedly in nutritional composition, food matrix, and processing, leading to contrasting effects on energy balance and adiposity.^26^^,^^27^ The protective associations of plant-sourced non-UPF with lower BMI and WC gain may reflect preservation of the natural food matrix, which is rich in fibre and bioactive compounds that enhance satiety, glycaemic control, gut microbiota function, and long-term energy balance.^14^^,^^15^

In contrast, plant-sourced UPF are typically energy-dense and high in added sugars, sodium, and saturated fats, promoting palatability and overconsumption.^14^^,^^15^ Processing disrupts the food matrix, reduces fibre, and alters satiety signalling and host–microbiota interactions.^28^ In addition, textural properties and flavour-enhancing additives may accelerate eating rates, reduce the number of chews per calorie and override appetite regulation,^15^^,^^16^^,^^29^ while other additives and processing-related contaminants have been linked to inflammation, metabolic dysfunction, and gut dysbiosis, all of which may contribute to weight and central fat gain.^14^^,^^15^

When dietary contributions were assessed using grams instead of energy, the total contribution of plant-sourced foods, which was not significant in the main analysis (% energy), showed an inverse association with BMI and WC increase. This may be explained by the lower energy density and higher volume of plant-sourced non-UPF, which are more evident when expressed as a proportion of total grams consumed. The consistent associations observed for plant-sourced UPF and non-UPF across both metrics, with stronger magnitudes in the gram-based analysis, indicate that gram-based indicators can complement energy-based measures by better reflecting the role of food volume and energy density in weight gain.

Key strengths include the large prospective cohort, repeated validated 24-h dietary recalls, and use of the standardized Nova classification^30^ enhancing robustness, comparability, and interpretability of the findings.

Some limitations should be acknowledged. Dietary intake may have been underreported, particularly for unhealthy foods such as plant-sourced UPF rich in added sugars or refined carbohydrates, potentially leading to underestimation of their dietary contribution and attenuation of associations with BMI and WC gain. This bias was partly mitigated by the self-administered online format, exclusion of implausible energy intakes, and use of repeated 24-h recalls. Misclassification of food due to limited information on food processing and food origin, particularly for composite dishes, is also possible. However, items were classified using established UK food data and standardized criteria, and any resulting misclassification is likely to be non-differential, which may have attenuated the observed associations. We could not account for dietary changes during follow-up, although prior evidence suggests that considering longitudinal dietary changes may strengthen observed associations.^31^ Residual confounding cannot be excluded despite extensive adjustment, and reverse causation is unlikely given the prospective design and sensitivity analyses excluding follow-up <2 years.

A further limitation relates to the temporal context of our data (2009–2012), which predates substantial changes in the plant-origin food market. Over the past decade, there has been a marked expansion in the availability and diversity of industrially processed plant-based products, including textured vegetable proteins such as vegetarian sausages, burgers, and soy-based meat substitutes (e.g., mince), as well as a rapid proliferation of convenience foods. More recently, retailers and fast-food chains have significantly increased their offerings of ready-to-eat and ready-to-heat plant-based meals, contributing to an exponential growth in this segment of the food environment.^32^ We did not account for potential temporal fluctuations in BMI and WC across follow-up assessments, as outcomes were defined based on the first observed increase from baseline. Another limitation of this study is that, although available, direct measures of body fat were not included in the present analyses, as their availability is more limited and less consistent across repeated assessments in this cohort. However, increases in BMI and WC are widely used and well-established indicators of adiposity and have been found consistently associated with higher cardiometabolic risk and adverse health outcomes in epidemiological studies.^33^^,^^34^ Finally, although cohort characteristics and effect sizes are broadly comparable to the UK population, generalizability to other populations should be interpreted with caution.^35^

Another consideration is the role of alcoholic beverages in dietary assessment. In this cohort, beer and wine (classified as plant-sourced non-UPF) contributed around 6% of total energy intake, while other alcoholic drinks (classified as plant-sourced UPF) represented only 0.7%. Although their inclusion could theoretically attenuate the protective association observed for plant-sourced non-UPF, sensitivity analyses excluding alcoholic beverages produced similar results, indicating that our findings were not driven by their inclusion.

A methodological limitation relates to the timing of outcome assessment. As the exact timing of BMI and WC increase was not directly observed but inferred between visits, the use of Cox models may introduce some imprecision in event timing, which could affect HR estimates. An additional consideration is the categorisation of continuous outcomes, which may lead to loss of information, although this approach was adopted to enhance clinical interpretability. The use of predefined thresholds (≥5% and ≥10%) is also baseline-dependent and may introduce heterogeneity not fully captured by adjustment for baseline BMI and WC.

Additionally, the substitution analysis is inherently model-dependent and may not fully account for the compositional and interdependent nature of dietary data; however, this parameterisation is consistent with approaches commonly used in previous nutritional epidemiology studies to assess relative dietary substitutions.^15^^,^^36^

Our findings support recommendations to reduce UPF consumption. Higher intake of plant-sourced non-UPF was associated with lower risk of BMI and WC increase, highlighting that the benefits of plant-based diets depend on minimizing food processing rather than simply increasing plant-derived foods. These results underscore that plant-based dietary recommendations should emphasize minimally processed foods and limit all UPF. Future research is needed to clarify the behavioural and biological mechanisms linking minimally processed diets to the prevention of weight gain and abdominal obesity.

## Contributors

PFH: Writing—Original draft; AGFG: Writing—Review & Editing; KC: Writing—Review & Editing; EPV: Writing—Review & Editing; RBL: Conceptualization, methodology, formal analysis and writing—review & editing; FR: Conceptualization, methodology, formal analysis and writing—original draft. PFH and FR accessed and verified the underlying data.

## Data sharing statement

The data supporting the findings of this study are available from the corresponding author upon reasonable request, subject to ethical and data protection restrictions.

## Declaration of interests

We declare no competing interests.
