# Supplementary material for: The association of plant or animal food origin and processing level with BMI and waist circumference: a prospective cohort study of the UK Biobank
Source: eClinicalMedicine. 2026 Jul 2;97:104050. doi: 10.1016/j.eclinm.2026.104050 (PMC13351747; doi:10.1016/j.eclinm.2026.104050)
Supplement: Supplementary Fig. S1 and Tables S1–S9 [file mmc1.pdf]

Manuscript title: The association of plant or animal food origin and processing level with BMI and waist circumference: a prospective cohort study of the UK Biobank

**Supplementary Table S1. Examples of food items considered in each food group.**	
**Plant-sourced foods**	
**Non-ultra-processed food**	
Fruit	Fresh, squeezed, chilled, frozen, or dried fruits (e.g., banana, orange, raisins); stewed/cooked fruit (e.g. apple, rhubarb, plums); Fruit juice fresh, smoothies, UHT or pasteurised.
Beer and Wine	Beer, lager, or cider; Red, rose or white wine (include sparkling).
Cereals	Grains such as brown, parboiled, or white rice, couscous, oat and other cooked grains such as bulgur wheat, millet or pearl barley
Vegetables	Fresh, chilled or frozen vegetables (e.g. broccoli, cabbage, carrots, cucumber, lettuce, spinach, tomatoes).
Pasta	White and wholemeal pasta; Noodles.
Roots and tubers	Starchy roots and tubers such as potatoes, sweet potatoes, and cassava;
Processed bread	Baguette, ciabatta, paninil; Oat cakes.
Nuts and seeds	Peanuts; unsalted, roasted or salted nuts (e.g. almonds, cashews, walnuts); Seeds (e.g. sunflower, pumpkin, linseeds)
Table sugar	Sugar; Honey; Syrup.
Vegetables/fruit preserved	Olives; Pickle; Sweetcorn (tinned); Jam.
Legumes	Beans (kidney beans/chickpeas/butter beans etc) or lentils; Hummus.
Others	Coffee/tea, Fungi, Soup without meat, vegetarian sushi, Plant oil
**Ultra-processed food**	
Industrialised packaged breads	Slice bread, Bread roll, bap, burger bun, hotdog roll, bagel; Crackers, crispbread, rice cakes, corn cakes
Pastries, buns, and cakes	Double or single crust pie/flan; Pancake, crêpe; Yorkshire pudding; Croissant; Scone (plain, fruit, cheese); Fruit cake, Cake, muffin, flapjack, brownie; Doughnuts; Sponge pudding; Cheesecake.
Biscuits	Chocolate covered biscuits; Chocolate biscuits; Sweet biscuits; Cereal bars manufactured.
Margarine and other spreads	Olive based spread; Margarine; Chocolate/nut spread
Industrial chips (French fries)	Potatoes (fried, chips, wedges, roast)
Confectionery	Chocolate bars; Chocolate sweets; Low sugar / sugar free sweets (hard and soft); Sweets (hard and soft, e.g. peppermints, toffees, fudge, fruit flavoured sweets)
Breakfast cereals	Sweetened oat crunch type cereal; extruded plain cereals with sugars.
Soft drinks, fruit drinks, and fruit juices	Fruit drinks and fruit juices, soft drinks, other beverages, Low calories drinks (soft or fruit drinks)
Packaged salty snacks	Crisps; Savoury crispbread/corn cake snacks; Cheesy biscuits.
Industrial pizza	All type.
Packaged pre-prepared meals	Dried/powdered soup; Carton/pouch/canned soup (pea, bean, lentil, vegetables, pasta); Snack pot, noodles/rice; baked beans.
Alcoholic drink	Spirits (e.g. vodka, whisky, gin, rum); other alcoholic drinks (e.g. Punch).
Sauces, dressing and gravies	Yeast extract; Tomato ketchup; Brown sauce/BBQ sauce; Salad dressing; Tomato-based sauce (e.g. pasta sauce); Gravy.
Meat alternatives	Vegetarian sausage/burger; Tofu/tempeh/TVP/soya mince; Quorn
**Animal -based foods**	
**Non-ultra-processed food**	
Red meat	Beef (e.g. roast, steak, mince, curry, burger); Pork (e.g. roast, chops, sweet and sour); Lamb or mutton (e.g. roast, chops, stew, burger);
Milk	Cow's milk; Goat's or sheep's milk; power milk; Cream (e.g. single, double, sour, crème fraiche)
Fish	Oily fish (e.g. salmon, tinned salmon, herring, mackerel, sardines, fresh tuna steak); White fish (e.g. cod, haddock, fish pie); Prawns, Lobster or crab; Shellfish (e.g. mussels, scallops); Tinned tuna.
Cheese	Low fat hard cheese; Hard cheese; Soft cheese; Blue cheese; Cottage cheese; Feta; Mozzarella; Goat's cheese.
Poultry	Chicken or turkey (e.g. roast, drumsticks, curry).
Animal fats	Butter; Ghee, dripping.
Eggs	Whole eggs (e.g. fried, boiled, poached); Omelettes or scrambled egg; Egg in sandwiches; Scotch egg.
Milk-based drinks	Dairy/yogurt-based smoothie; Yogurt; Flavoured milk or milkshakes; Hot chocolate (including low calorie).
**Ultra-processed food**	
Sausage and other reconstituted red meat products	Sausage; Ham/Parma ham/salami/pastrami/cured meats.
Nuggets and other reconstituted meat products	Chicken or turkey in breadcrumbs or deep fried; Liver or liver pâté; Breaded fish (e.g. fish fingers) or fish cakes; Battered fish.
Milk based desserts	Ice-cream; Custard, rice pudding; Other milk-based desserts (e.g. mousse, tiramisu, crème caramel)
Mayonnaise and spreadable cheese	Mayonnaise/salad cream (including low fat); Cheese sauce (e.g. cauliflower cheese); White sauce/cream sauce (e.g. bechamel)

Supplementary Table S2. Test for linearity assumption of the association between the food groups and BMI or waist circumference increase, using restricted cubic spline functions.

Food groups	≥5% BMI increase	≥10% BMI increase	≥5% WC increase	≥10% WC increase
		P value		
Plant-sourced non-UPF	0·488	0·678	0·828	0·767
Plant-sourced UPF	0·636	0·465	0·957	0·851
All plant-sourced foods	0·876	0·870	0·300	0·382
All UPF	0·589	0·912	0·783	0·639

BMI = Body Mass Index; WC= waist circumference; UPF = ultra-processed.

Figure S1. Restricted cubic spline plots of the association between food group intake and the risk of BMI and waist circumference increase

S1a)

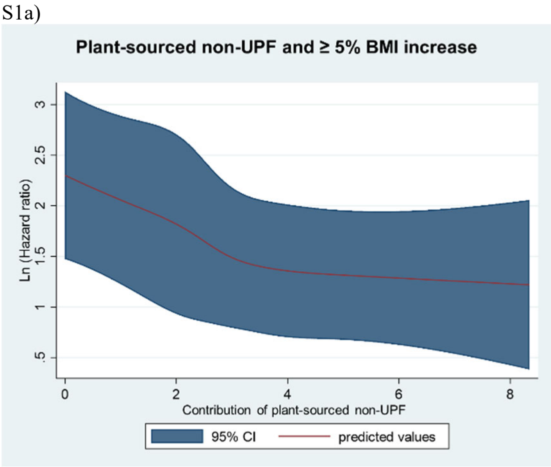

S1b)

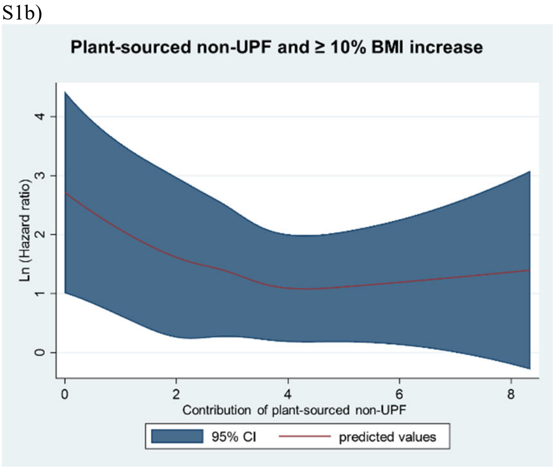

S1c)

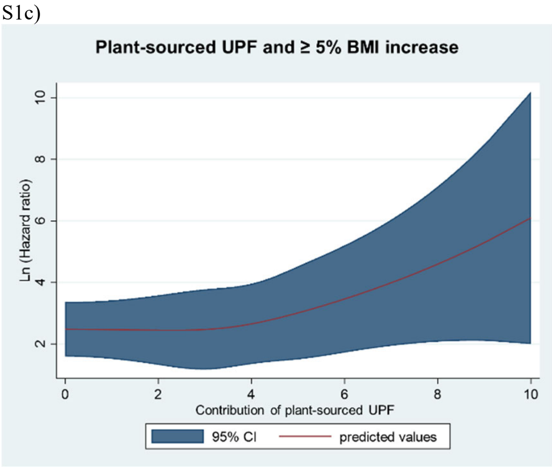

S1d)

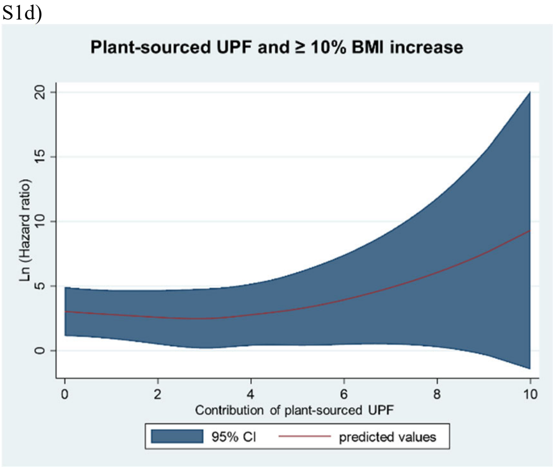

S1e)

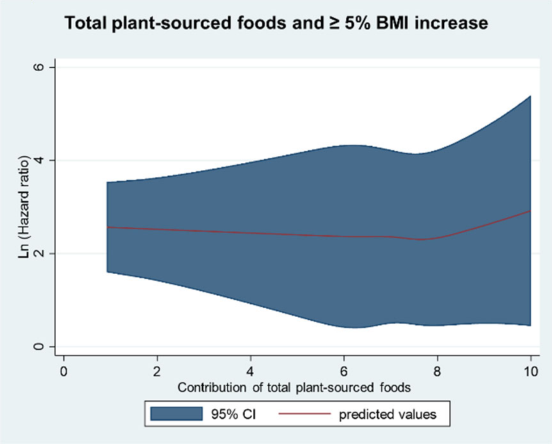

S1f)

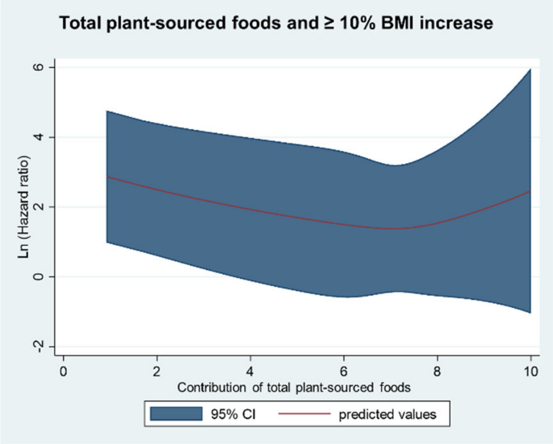

S1g)

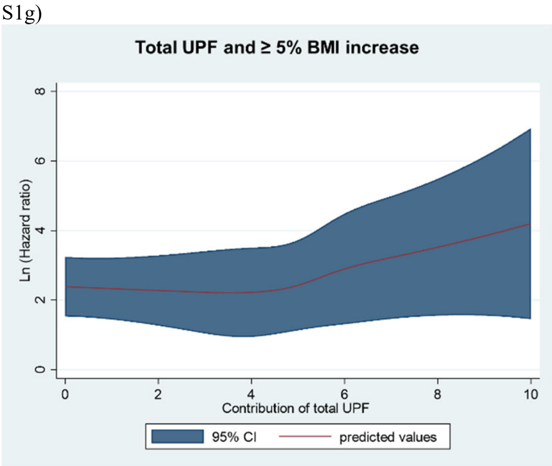

S1h)

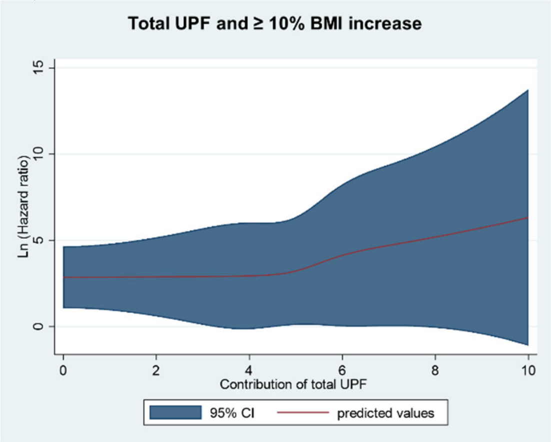

S1i)

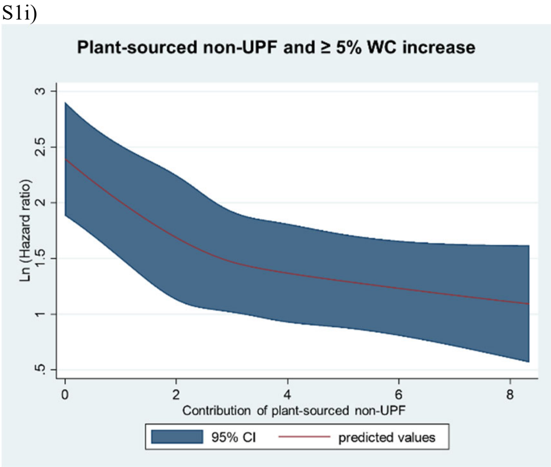

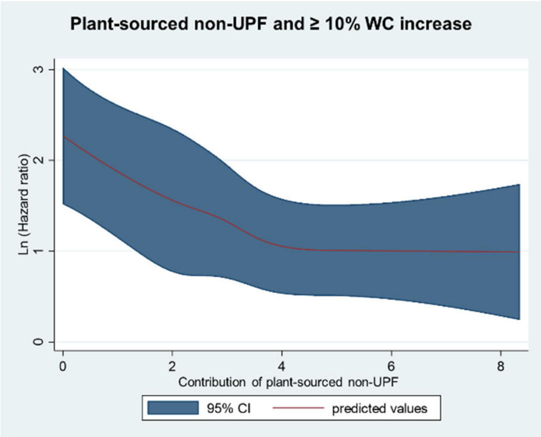

S1j)

S1k)

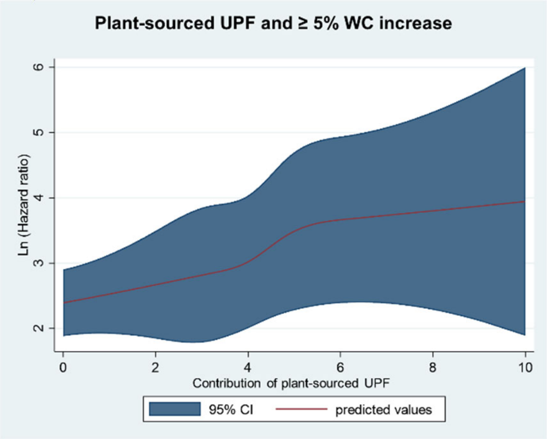

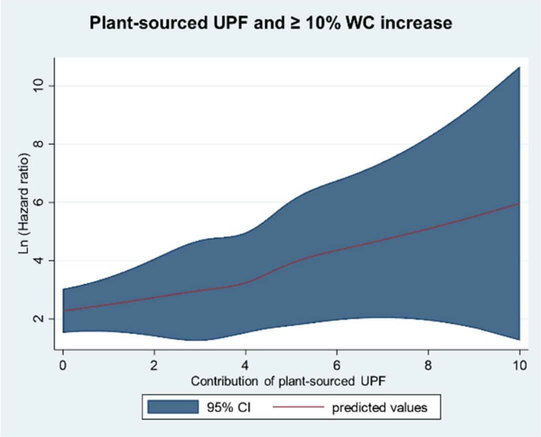

S1l)

S1m)

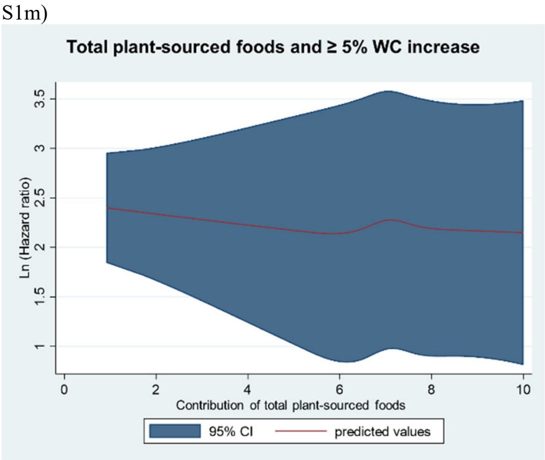

S1n)

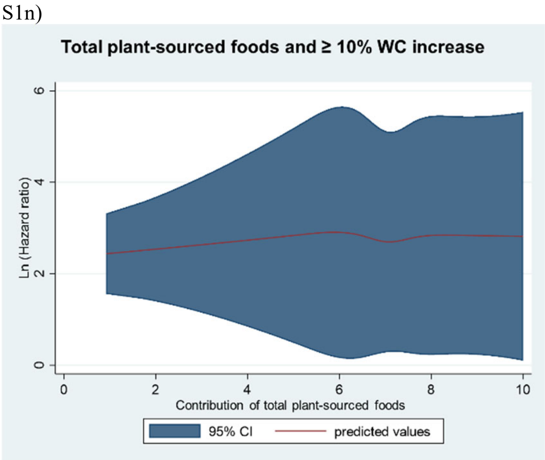

S1o)

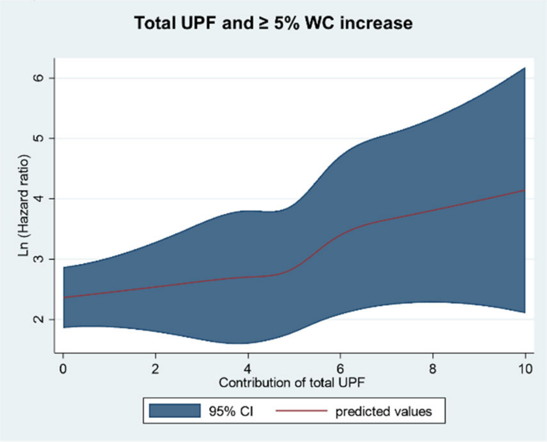

S1p)

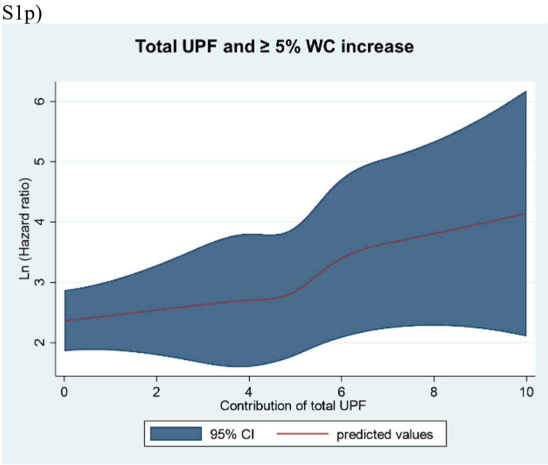

Supplementary Table S3. Sensitivity analysis for the association between the consumption of foods groups, and BMI or waist circumference increase, considering the dietary contribution of total grams in the UK Biobank cohort (n = 17·374).

	**Consumption in grams (% of total grams)**													
	**Quartile** [a]											**Continuous (10% increase in the consumption)**		
	**1**	**2**			**3**			**4**						
	HR (95% CI)										*p for trend*[a]	*HR (95% CI)*		
**For having a ≥ 5% BMI increase**														
n for cases/non-cases = 2477/14897														
Plant-sourced non-UPF	**1**	**0·78**	0·70	0·87	**0·71**	0·64	0·79	**0·61**	0·55	0·69	*< 0·001*	**0·87**	0·85	0·90
Plant-sourced UPF	**1**	**1·20**	1·07	1·36	**1·27**	1·13	1·43	**1·70**	1·51	1·91	*< 0·001*	**1·20**	1·17	1·25
Total Plant-sourced	**1**	**0·85**	0·76	0·95	**0·83**	0·74	0·92	**0·91**	0·81	1·01	*0·055*	**0·94**	0·90	0·99
Total UPF	**1**	**1·26**	1·12	1·42	**1·24**	1·10	1·41	**1·74**	1·55	1·95	*< 0·001*	**1·19**	1·15	1·22
**For having a ≥ 10% BMI increase**														
n for cases/non-cases =737/16637														
Plant-sourced non-UPF	**1**	**0·70**	0·58	0·85	**0·62**	0·51	0·76	**0·45**	0·36	0·56	*< 0·001*	**0·82**	0·78	0·86
Plant-sourced UPF	**1**	**1·41**	1·11	1·79	**1·59**	1·26	2·01	**2·38**	1·90	2·96	*< 0·001*	**1·30**	1·23	1·38
Total Plant-sourced	**1**	**0·80**	0·65	0·98	**0·68**	0·55	0·84	**0·95**	0·78	1·15	*0·299*	**0·92**	0·84	0·99
Total UPF	**1**	**1·53**	1·21	1·94	**1·47**	1·16	1·87	**2·50**	2·00	3·13	*< 0·001*	**1·28**	1·22	1·35
**For having a ≥ 5% WC increase**														
n for cases/non-cases = 4567/12807														
Plant-sourced non-UPF	**1**	**0·87**	0·80	0·95	**0·81**	0·74	0·88	**0·70**	0·64	0·76	*< 0·001*	**0·90**	0·89	0·92
Plant-sourced UPF	**1**	**1·21**	1·11	1·32	**1·34**	1·23	1·46	**1·52**	1·39	1·66	*< 0·001*	**1·16**	1·13	1·19
Total Plant-sourced	**1**	**0·96**	0·88	1·05	**0·89**	0·82	0·97	**0·95**	0·88	1·04	*0·099*	**0·96**	0·92	0·99
Total UPF	**1**	**1·19**	1·09	1·30	**1·26**	1·16	1·38	**1·51**	1·39	1·65	*< 0·001*	**1·14**	1·11	1·17
**For having a ≥ 10% WC increase**														
n for cases /non-cases =1908/15466														
Plant-sourced non-UPF	**1**	**0·77**	0·68	0·88	**0·70**	0·62	0·80	**0·59**	0·52	0·68	*< 0·001*	**0·86**	0·83	0·89
Plant-sourced UPF	**1**	**1·32**	1·15	1·52	**1·39**	1·21	1·59	**1·83**	1·59	2·10	*< 0·001*	**1·24**	1·19	1·29
Total Plant-sourced	**1**	**0·92**	0·80	1·04	**0·86**	0·75	0·98	**0·91**	0·79	1·03	*0·084*	**0·93**	0·88	0·99
Total UPF	**1**	**1·30**	1·14	1·50	**1·35**	1·17	1·55	**1·87**	1·63	2·14	*< 0·001*	**1·21**	1·16	1·25

BMI = Body Mass Index; WC= waist circumference; UPF = ultra-processed foods

Mean follow-up times were 5·3 for BMI increase (92·129·583 person-years) and for WC increase (92·135·083 person-years).

Cut-offs for quarters of food consumption ranged from 45·7% (1st quartile) to 79·5% (4th quartile) for plant-based foods non-UPF; from 7·2% to 32·3% for plant-based UPF; from 71.5% to 90.9% for total plant-based foods; and 10·3% to 38·3% for UPF, respectively.

Cox proportional hazards models with age as the underlying timescale. Adjusted by sex, ethnic (white, non-white), baseline BMI or WC (continuous, depending on the outcome), physical activity (low, moderate, high, missing), smoking status (never, previous, current), index of multiple deprivation (quintile), region (London, South East, South West, East Midlands, West Midlands, Yorkshire & the Humber, North East, North West, Wales, Scotland), and total energy intake. Analysis were stratified by sex in both outcomes and for baseline WC in relation to WC gain.

Supplementary Table S4. Sensitivity analysis for the association between the consumption of foods groups, and BMI or waist circumference increase, additionally adjustment for nutrients (free sugars, saturated fats, sodium and fiber) in the UK Biobank cohort (n = 17·374).

	Consumption (% of total energy)													
	Quartile [a]											Continuous (10% increase in the consumption)		
	1	2			3			4						
					HR (95% CI)						*p for trend*[a]	*HR (95% CI)*		
**For having a ≥ 5% BMI increase**														
n for cases/non-cases = 2477/14897														
Plant-sourced non-UPF	**1**	**0·88**	0·79	0·99	**0·81**	0·72	0·91	**0·78**	0·68	0·89	*< 0·001*	**0·92**	0·88	0·96
Plant-sourced UPF	**1**	**1·01**	0·90	1·13	**1·07**	0·95	1·20	**1·18**	1·05	1·33	*0·004*	**1·06**	1·02	1·09
Total Plant-sourced	**1**	**1·05**	0·94	1·17	**1·01**	0·90	1·13	**1·03**	0·91	1·16	*0·794*	**1·02**	0·97	1·06
Total UPF	**1**	**0·95**	0·85	1·08	**1·06**	0·94	1·20	**1·14**	1·00	1·30	*0·018*	**1·04**	1·00	1·08
**For having a ≥ 10% BMI increase**														
n for cases/non-cases =737/16637														
Plant-sourced non-UPF	**1**	**0·86**	0·71	1·05	**0·72**	0·57	0·89	**0·70**	0·54	0·89	*0·001*	**0·88**	0·81	0·96
Plant-sourced UPF	**1**	**0·91**	0·73	1·14	**1·06**	0·86	1·31	**1·23**	0·99	1·52	*0·025*	**1·08**	1·01	1·14
Total Plant-sourced	**1**	**0·94**	0·77	1·16	**0·92**	0·75	1·14	**1·05**	0·85	1·29	*0·744*	**1·01**	0·93	1·08
Total UPF	**1**	**0·92**	0·73	1·15	**1·14**	0·91	1·42	**1·19**	0·93	1·51	*0·062*	**1·05**	0·98	1·12
**For having a ≥ 5% WC increase**														
n for cases/non-cases = 4567/12807														
Plant-sourced non-UPF	**1**	**0·92**	0·84	1·00	**0·85**	0·77	0·93	**0·80**	0·72	0·89	*< 0·001*	**0·92**	0·89	0·96
Plant-sourced UPF	**1**	**1·06**	0·97	1·15	**1·08**	0·99	1·18	**1·27**	1·15	1·39	*< 0·001*	**1·06**	1·03	1·09
Total Plant-sourced	**1**	**1·06**	0·97	1·15	**1·08**	0·99	1·18	**1·05**	0·96	1·16	*0·209*	**1·02**	0·99	1·05
Total UPF	**1**	**1·03**	0·94	1·12	**1·10**	1·01	1·21	**1·23**	1·11	1·36	*< 0·001*	**1·06**	1·03	1·09
**For having a ≥ 10% WC increase**														
n for cases /non-cases =1908/15466														
Plant-sourced non-UPF	**1**	**0·92**	0·81	1·05	**0·78**	0·68	0·90	**0·71**	0·60	0·83	*< 0·001*	**0·89**	0·84	0·94
Plant-sourced UPF	**1**	**1·05**	0·91	1·20	**1·09**	0·95	1·25	**1·34**	1·16	1·54	*< 0·001*	**1·08**	1·04	1·13
Total Plant-sourced	**1**	**1·05**	0·92	1·20	**1·02**	0·89	1·17	**1·06**	0·92	1·22	*0·486*	**1·02**	0·97	1·07
Total UPF	**1**	**0·95**	0·83	1·10	**1·13**	0·98	1·31	**1·23**	1·05	1·44	*0·002*	**1·07**	1·03	1·12

BMI = Body Mass Index; WC= waist circumference; UPF = ultra-processed foods

Mean follow-up times were 5·3 for BMI increase (92·129·583 person-years) and for WC increase (92·135·083 person-years).

Cut-offs for quarters of food consumption ranged from 16·3% (1st quartile) to 45·2% (4th quartile) for plant-based foods non-UPF; from 23·5% to 56·5% for plant-based UPF; from 56·6% to 82·0% for total plant-based foods; and 31·2% to 65·9% for UPF, respectively.

Cox proportional hazards models with age as the underlying timescale· Adjusted by sex, ethnic (white, non-white), baseline BMI or WC (continuous, depending on the outcome), physical activity (low, moderate, high, missing), smoking status (never, previous, current), index of multiple deprivation (quintile), and region (London, South East, South West, East Midlands, West Midlands, Yorkshire & the Humber, North East, North West, Wales, Scotland). Analysis were stratified by sex in both outcomes and for baseline WC in relation to WC gain.

Supplementary Table S5· Sensitivity analysis for the association between the consumption of foods groups, and BMI or waist circumference increase, excluding participants who were on a diet (n = 16·886).

	Consumption (% of total energy)													
	Quartile [a]											Continuous (10% increase in the consumption)		
	**1**	**2**			**3**			**4**						
		HR (95% CI)									*p for trend[a]*	*HR (95% CI)*		
**For having a ≥ 5% BMI increase**														
n for cases/non-cases =2361/14505														
Plant-sourced non-UPF	**1**	**0·84**	0·75	0·93	**0·76**	0·68	0·85	**0·69**	0·61	0·77	*< 0·001*	**0·89**	0·85	0·92
Plant-sourced UPF	**1**	**1·03**	0·92	1·16	**1·14**	1·01	1·28	**1·34**	1·19	1·50	*< 0·001*	**1·10**	1·07	1·14
Total Plant-sourced	**1**	**1·02**	0·91	1·14	**0·99**	0·88	1·11	**1·02**	0·91	1·14	*0·875*	**1·01**	0·97	1·05
Total UPF	**1**	**1·01**	0·89	1·13	**1·18**	1·05	1·33	**1·38**	1·23	1·55	*< 0·001*	**1·10**	1·07	1·13
**For having a ≥ 10% BMI increase**														
n for cases/non-cases =679/16207														
Plant-sourced non-UPF	**1**	**0·80**	0·66	0·98	**0·66**	0·54	0·82	**0·64**	0·52	0·80	*< 0·001*	**0·86**	0·80	0·92
Plant-sourced UPF	**1**	**0·95**	0·75	1·19	**1·14**	0·91	1·41	**1·47**	1·19	1·81	*< 0·001*	**1·14**	1·08	1·21
Total Plant-sourced	**1**	**0·91**	0·74	1·13	**0·94**	0·75	1·16	**1·12**	0·91	1·38	*0·276*	**1·03**	0·96	1·11
Total UPF	**1**	**0·99**	0·78	1·25	**1·37**	1·10	1·70	**1·55**	1·25	1·93	*< 0·001*	**1·14**	1·08	1·21
**For having a ≥ 5% WC increase**														
n for cases/non-cases = 4408/12478														
Plant-sourced non-UPF	**1**	**0·90**	0·83	0·98	**0·81**	0·74	0·88	**0·75**	0·69	0·82	*< 0·001*	**0·91**	0·88	0·93
Plant-sourced UPF	**1**	**1·08**	0·99	1·17	**1·12**	1·03	1·22	**1·37**	1·25	1·49	*< 0·001*	**1·08**	1·06	1·11
Total Plant-sourced	**1**	**1·03**	0·95	1·12	**1·06**	0·97	1·15	**1·04**	0·95	1·13	*0·313*	**1·01**	0·98	1·04
Total UPF	**1**	**1·06**	0·97	1·16	**1·17**	1·07	1·28	**1·36**	1·24	1·48	*< 0·001*	**1·09**	1·06	1·11
**For having a ≥ 10% WC increase**														
n for cases /non-cases =1836/15050														
Plant-sourced non-UPF	**1**	**0·88**	0·78	1·00	**0·72**	0·63	0·82	**0·63**	0·54	0·72	*< 0·001*	**0·85**	0·81	0·89
Plant-sourced UPF	**1**	**1·10**	0·96	1·27	**1·19**	1·03	1·37	**1·55**	1·35	1·78	*< 0·001*	**1·13**	1·09	1·17
Total Plant-sourced	**1**	**1·03**	0·90	1·18	**0·98**	0·86	1·13	**1·04**	0·91	1·19	*0·720*	**1·00**	0·95	1·05
Total UPF	**1**	**1·02**	0·88	1·17	**1·28**	1·12	1·47	**1·50**	1·31	1·72	*< 0·001*	**1·13**	1·09	1·17

BMI = Body Mass Index; WC= waist circumference; UPF = ultra-processed foods

Mean follow-up times were 5·3 for BMI increase (89·109·75 person-years) and for WC increase (89·115·25 person-years).

Cut-offs for quarters of food consumption ranged from 16·3% (1st quartile) to 45·1% (4th quartile) for plant-based foods non-UPF; from 23·7% to 56·6% for plant-based UPF; from 56·7% to 82·1% for total plant-based foods; and 31·3% to 66·0% for UPF, respectively.

Cox proportional hazards models with age as the underlying timescale· Adjusted by sex, ethnic (white, non-white), baseline BMI or WC (continuous, depending on the outcome), physical activity (low, moderate, high, missing), smoking status (never, previous, current), index of multiple deprivation (quintile), and region (London, South East, South West, East Midlands, West Midlands, Yorkshire & the Humber, North East, North West, Wales, Scotland)· Analysis were stratified by sex in both outcomes and for baseline WC in relation to WC gain.

Supplementary Table S6· Sensitivity analysis for the association between the consumption of foods groups, and BMI or waist circumference increase, disregarding alcoholic beverages consumption (n = 17·374)·

	Consumption (% of total energy)														
	Quartile [a]												Continuous (10% increase in the consumption)		
	**1**	**2**			**3**			**4**							
		HR (95% CI)										*p for trend*[a]	*HR (95% CI)*		
**For having a ≥ 5% BMI increase**															
n for cases/non-cases = 2477/14897															
Plant-sourced non-UPF	**1**	**0·88**	0·79	0·98	**0·78**	0·70	0·87	**0·66**	0·59	0·75		*< 0·001*	**0·87**	0·84	0·90
Plant-sourced UPF	**1**	**1·10**	0·98	1·23	**1·14**	1·02	1·28	**1·42**	1·27	1·59		*< 0·001*	**1·10**	1·07	1·13
Total Plant-sourced	**1**	**0·97**	0·87	1·08	**0·95**	0·85	1·06	**1·00**	0·89	1·12		*0·872*	**1·00**	0·97	1·04
Total UPF	**1**	**1·03**	0·92	1·16	**1·20**	1·07	1·34	**1·39**	1·24	1·55		*< 0·001*	**1·10**	1·07	1·14
**For having a ≥ 10% BMI increase**															
n for cases/non-cases =737/16637															
Plant-sourced non-UPF	**1**	**0·77**	0·63	0·93	**0·67**	0·55	0·82	**0·58**	0·47	0·72		*< 0·001*	**0·84**	0·78	0·90
Plant-sourced UPF	**1**	**1·02**	0·82	1·26	**1·10**	0·89	1·36	**1·48**	1·21	1·82		*< 0·001*	**1·13**	1·07	1·19
Total Plant-sourced	**1**	**0·94**	0·77	1·15	**0·87**	0·71	1·08	**1·06**	0·86	1·29		*0·771*	**1·01**	0·95	1·08
Total UPF	**1**	**1·02**	0·82	1·27	**1·31**	1·06	1·62	**1·54**	1·25	1·89		*< 0·001*	**1·14**	1·08	1·20
**For having a ≥ 5% WC increase**															
n for cases/non-cases = 4567/12807															
Plant-sourced non-UPF	**1**	**0·87**	0·80	0·94	**0·78**	0·72	0·85	**0·70**	0·64	0·76		*< 0·001*	**0·88**	0·86	0·91
Plant-sourced UPF	**1**	**1·12**	1·03	1·22	**1·18**	1·08	1·29	**1·40**	1·29	1·53		*< 0·001*	**1·09**	1·07	1·12
Total Plant-sourced	**1**	**1·02**	0·94	1·11	**1·03**	0·95	1·12	**1·00**	0·92	1·09		*0·945*	**1·00**	0·98	1·03
Total UPF	**1**	**1·07**	0·98	1·17	**1·22**	1·12	1·33	**1·41**	1·29	1·54		*< 0·001*	**1·10**	1·08	1·13
**For having a ≥ 10% WC increase**															
n for cases /non-cases =1908/15466															
Plant-sourced non-UPF	**1**	**0·85**	0·75	0·97	**0·74**	0·65	0·85	**0·58**	0·51	0·67		*< 0·001*	**0·83**	0·79	0·87
Plant-sourced UPF	**1**	**1·16**	1·02	1·33	**1·24**	1·08	1·42	**1·57**	1·37	1·80		*< 0·001*	**1·13**	1·09	1·18
Total Plant-sourced	**1**	**1·00**	0·87	1·14	**0·94**	0·82	1·07	**1·00**	0·88	1·14		*0·762*	**1·00**	0·96	1·04
Total UPF	**1**	**1·16**	1·01	1·33	**1·31**	1·14	1·50	**1·62**	1·42	1·86		*< 0·001*	**1·14**	1·10	1·18

BMI = Body Mass Index; WC= waist circumference; UPF = ultra-processed foods

Mean follow-up times were 5·3 for BMI increase (92·129·583 person-years) and for WC increase (92·135·083 person-years).

Cut-offs for quarters of food consumption ranged from 13·0% (1st quartile) to 40·5% (4th quartile) for plant-based foods non-UPF; from 25·0% to 58·7% for plant-based UPF; from 53·6% to 80·6% for total plant-based foods; and 33·6% to 68·2% for UPF, respectively.

Cox proportional hazards models with age as the underlying timescale· Adjusted by sex, ethnic (white, non-white), baseline BMI or WC (continuous, depending on the outcome), physical activity (low, moderate, high, missing), smoking status (never, previous, current), index of multiple deprivation (quintile), and region (London, South East, South West, East Midlands, West Midlands, Yorkshire & the Humber, North East, North West, Wales, Scotland)· Analysis were stratified by sex in both outcomes and for baseline WC in relation to WC gain.

Supplementary Table S7· Sensitivity analysis for the association between the consumption of foods groups, and BMI or waist circumference increase, excluding participants with severe obesity (n = 17·206)·

	Consumption (% of total energy)													
	Quartile [a]											Continuous (10% increase in the consumption)		
	1	2			3			4						
					HR (95% CI)						*p for trend*[a]	*HR (95% CI)*		
**For having a ≥ 5% BMI increase**														
n for cases/non-cases = 2450/14756														
Plant-sourced non-UPF	**1**	**0·85**	0·76	0·94	**0·75**	0·67	0·84	**0,69**	0·62	0·78	*< 0·001*	**0·89**	0·85	0·92
Plant-sourced UPF	**1**	**1·05**	1.03	1·29	**1·15**	1·03	1·30	**1·34**	1·20	1·50	*< 0·001*	**1·10**	1·07	1·13
Total Plant-sourced	**1**	**1.01**	0·91	1·13	**0·98**	0·88	1·10	**1·02**	0·91	1·14	*0·842*	**1·00**	0·97	1·04
Total UPF	**1**	**1·02**	0·91	1·15	**1·19**	1·06	1·33	**1·38**	1·23	1·54	*< 0·001*	**1·10**	1·07	1·13
**For having a ≥ 10% BMI increase**														
n for cases/non-cases =724/16482														
Plant-sourced non-UPF	**1**	**0·82**	0·68	0·99	**0·66**	0·54	0·82	**0·62**	0·50	0·76	*< 0·001*	**0·85**	0·79	0·91
Plant-sourced UPF	**1**	**0.97**	0·77	1·20	**1·18**	0·95	1·46	**1·45**	1·18	1·78	*< 0·001*	**1·13**	1·07	1·20
Total Plant-sourced	**1**	**0·90**	0·73	1·11	**0·89**	0·72	1·10	**1·06**	0·87	1·30	*0·607*	**1·00**	0·94	1·08
Total UPF	**1**	**1·01**	0·81	1·27	**1·34**	1·08	1·66	**1·55**	1·26	1·91	*< 0·001*	**1·14**	1·08	1·20
**For having a ≥ 5% WC increase**														
n for cases/non-cases = 4528/12678														
Plant-sourced non-UPF	**1**	**0·89**	0·82	0·97	**0·80**	0·74	0·88	**0·74**	0·68	0·71	*< 0·001*	**0·91**	0·88	0·93
Plant-sourced UPF	**1**	**1·09**	1·00	1·19	**1·14**	1·04	1·24	**1·37**	1·25	1·49	*< 0·001*	**1·09**	1·06	1·11
Total Plant-sourced	**1**	**1·02**	0·94	1·11	**1·05**	0·96	1·14	**1·03**	0·94	1·11	*0·466*	**1·00**	0·98	1·04
Total UPF	**1**	**1·07**	0·98	1·17	**1·19**	1·09	1·29	**1·37**	1·25	1·49	*< 0·001*	**1·09**	1·06	1·11
**For having a ≥ 10% WC increase**														
n for cases /non-cases =1889/15317														
Plant-sourced non-UPF	**1**	**0·89**	0·78	1.01	**0·72**	0·64	0·83	**0·62**	0·54	0·71	*< 0·001*	**0·85**	0·81	0·89
Plant-sourced UPF	**1**	**1·11**	0.97	1·27	**1·21**	1·04	1·37	**1·55**	1·36	1·77	*< 0·001*	**1·13**	1·09	1·17
Total Plant-sourced	**1**	**1·01**	0·88	1·15	**1.00**	0·87	1·14	**1·04**	0·91	1·18	*0·665*	**1·00**	0·96	1·04
Total UPF	**1**	**1·03**	0.90	1·19	**1·29**	1·13	1·48	**1·50**	1·31	1·71	*< 0·001*	**1·13**	1·09	1·17

BMI = Body Mass Index; WC= waist circumference; UPF = ultra-processed foods

Mean follow-up times were 5·3 for BMI increase (91 306 583person-years) and for WC increase (91 312.083 person-years).

Cox proportional hazards models with age as the underlying timescale· Adjusted by sex, ethnic (white, non-white), baseline BMI or WC (continuous, depending on the outcome), physical activity (low, moderate, high, missing), smoking status (never, previous, current), index of multiple deprivation (quintile), and region (London, South East, South West, East Midlands, West Midlands, Yorkshire & the Humber, North East, North West, Wales, Scotland)·

Supplementary Table S8· Sensitivity analysis for the association between the consumption of foods groups, and BMI or waist circumference increase, excluding participants with diabetes (n = 16 854)·

**Consumption (% of total energy)**	**Quartile [a]**											**Continuous (10% increase in the consumption)**		
	**1**	**2**			**3**			**4**						
	HR (95% CI)										*p for trend[α]*	*HR (95% CI)*		
**For having a ≥ 5% BMI increase**														
n for cases/non-cases = 2413/14441														
Plant-sourced non-UPF	**1**	**0·85**	0·76	0·94	**0·74**	0·66	0·83	**0,70**	0·62	0·78	*< 0·001*	**0·89**	0·85	0·92
Plant-sourced UPF	**1**	**1·05**	0.93	1·18	**1·15**	1·02	1·29	**1·34**	1·20	1·51	*< 0·001*	**1·10**	1·06	1·13
Total Plant-sourced	**1**	**1.01**	0·91	1·13	**0·98**	0·88	1·10	**1·02**	0·91	1·14	*0·882*	**1·00**	0·96	1·04
Total UPF	**1**	**1·02**	0·90	1·14	**1·19**	1·06	1·34	**1·38**	1·23	1·55	*< 0·001*	**1·10**	1·07	1·13
**For having a ≥ 10% BMI increase**														
n for cases/non-cases =719/16135														
Plant-sourced non-UPF	**1**	**0·83**	0·69	1.00	**0·66**	0·54	0·82	**0·62**	0·50	0·77	*< 0·001*	**0·85**	0·80	0·91
Plant-sourced UPF	**1**	**0.96**	0·77	1·20	**1·18**	0·96	1·47	**1·45**	1·18	1·79	*< 0·001*	**1·13**	1·07	1·20
Total Plant-sourced	**1**	**0·93**	0·75	1·14	**0·90**	0·73	1·11	**1·08**	0·88	1·33	*0·523*	**1·00**	0·94	1·08
Total UPF	**1**	**1·02**	0·82	1·28	**1·37**	1·11	1·70	**1·55**	1·26	1·92	*< 0·001*	**1·14**	1·08	1·20
**For having a ≥ 5% WC increase**														
n for cases/non-cases = 4450/12404														
Plant-sourced non-UPF	**1**	**0·90**	0·83	0·98	**0·81**	0·74	0·88	**0·74**	0·68	0·81	*< 0·001*	**0·90**	0·88	0·93
Plant-sourced UPF	**1**	**1·09**	1·00	1·19	**1·13**	1·03	1·23	**1·37**	1·26	1·49	*< 0·001*	**1·09**	1·06	1·11
Total Plant-sourced	**1**	**1·02**	0·93	1·11	**1·05**	0·96	1·14	**1·03**	0·94	1·12	*0·404*	**1·00**	0·98	1·04
Total UPF	**1**	**1·07**	0·98	1·17	**1·18**	1·08	1·29	**1·37**	1·25	1·49	*< 0·001*	**1·09**	1·06	1·11
**For having a ≥ 10% WC increase**														
n for cases /non-cases =1866/14988														
Plant-sourced non-UPF	**1**	**0·88**	0·77	1.00	**0·72**	0·63	0·82	**0·63**	0·55	0·72	*< 0·001*	**0·85**	0·82	0·89
Plant-sourced UPF	**1**	**1·11**	0.97	1·27	**1·16**	1·01	1·33	**1·55**	1·35	1·77	*< 0·001*	**1·13**	1·09	1·17
Total Plant-sourced	**1**	**1·01**	0·88	1·15	**0.98**	0·86	1·12	**1·04**	0·91	1·19	*0·681*	**1·00**	0·95	1·05
Total UPF	**1**	**1·00**	0.88	1·16	**1·26**	1·10	1·44	**1·48**	1·30	1·70	*< 0·001*	**1·13**	1·09	1·17

BMI = Body Mass Index; WC= waist circumference; UPF = ultra-processed foods

Mean follow-up times were 5·3 for BMI increase (89 549.583 person-years) and for WC increase (89 555.083 person-years).

Cox proportional hazards models with age as the underlying timescale· Adjusted by sex, ethnic (white, non-white), baseline BMI or WC (continuous, depending on the outcome), physical activity (low, moderate, high, missing), smoking status (never, previous, current), index of multiple deprivation (quintile), and region (London, South East, South West, East Midlands, West Midlands, Yorkshire & the Humber, North East, North West, Wales, Scotland)·

Suplementary Table S9. Sensitivity analysis for the association between the consumption of foods groups, and weight gain or waist circumference increase, interaction between sex (n = 17,374).

	Consumption (% of total energy)									
	Quartile [a]									
	**1**	**2**			**3**			**4**		
					HR (95% CI)					
**For having a ≥ 5% BMI increase**										
n for cases/non-cases =2477/14897										
Plant-sourced non-UPF	**1**	**0.84**	0.75	0.94	**0.74**	0.66	0.84	**0.67**	0.59	0.78
Plant-sourced UPF	**1**	**1.06**	0.94	1.19	**1.16**	1.03	1.32	**1.36**	1.18	1.56
**For having a ≥ 10% BMI increase**										
n for cases/non-cases =737/16637										
Plant-sourced non-UPF	**1**	**0.82**	0.68	1.00	**0.65**	0.52	0.81	**0.61**	0.48	0.78
Plant-sourced UPF	**1**	**0.97**	0.78	1.21	**1.18**	0.94	1.47	**1.46**	1.15	1.85
**For having a ≥ 5% WC increase**										
n for cases/non-cases = 4567/12807										
Plant-sourced non-UPF	**1**	**0.89**	0.81	0.97	**0.80**	0.73	0.88	**0.73**	0.66	0.82
Plant-sourced UPF	**1**	**1.09**	1.00	1.19	**1.14**	1.04	1.25	**1.37**	1.23	1.53
**For having a ≥ 10% WC increase**										
n for cases /non-cases =1908/15466										
Plant-sourced non-UPF	**1**	**0.88**	0.77	1.00	**0.71**	0.62	0.82	**0.61**	0.52	0.72
Plant-sourced UPF	**1**	**1.12**	0.98	1.29	**1.22**	1.05	1.41	**1.61**	1.37	1.89
